# Optimization of Cyber Tactics in Sports Strategies Using Hybrid AI Decision-Making Technologies

**DOI:** 10.1155/2022/3762755

**Published:** 2022-06-13

**Authors:** Meiling Duan

**Affiliations:** Zhengzhou Preschool Education College, Zhengzhou 450000, China

## Abstract

One of the main problems of modern research concerns the optimal design of solutions to address cybersecurity problems, with methods that provide the ability to choose an objective function different from that of the classic problem of more economical design while allowing the use of constraints on any possible variable during planning, including financial resources. The number of corresponding solutions concerns the issue of optimal design of security strategies with a simulation that lies in game theory, with players, the defender on one side defending the system managing the available options of strategic solutions, and the attacker, who chooses the way to strike the system, based on some attack scenarios that cannot be easily predicted. The inherent difficulty of implementing the proposed solutions lies in the combined explosion of all possible combinations that make up the solution space, the complete examination of which requires a lot of computational time and computing resources, to the point that their use becomes unprofitable. This weakness is attributed to even minor problems, and the possible strategies available to the defender are finite but at the same time numerous. To solve the abovementioned problem, the work proposes a hybrid system that aims to identify the best possible approach in the theoretically optimal solution in a short time and with minimal computing resources. Specifically, a heuristic optimization methodology is used with overlapping answers between two contiguous neighborhoods based on the Bloom Filters structure that supports fast listings and searches. This methodology, which is evaluated in optimizing safety strategies in the sports industry, brings about 40% optimization.

## 1. Introduction

Game theory is the branch that deals with the analysis and evaluation of games if players behave logically. This definition distinguishes the term “game,” which is most often used in everyday life and has mainly a recreational meaning. A game is any situation in which two or more people, called players, are called upon to make one or more decisions, depending on which event will occur, which has a different value for each player [1].

The definition of *utility* is perhaps one of the most fundamental concepts in game theory [2], as it dramatically facilitates their handling and analysis. It was designed to determine a person's level of contentment as a consequence of a particular outcome [3, 4]. More specifically, utility is an arbitrary measure of satisfaction that aims to quantify the effect of an event on a person's happiness. Like any size, the utility has its unit of measurement, the *util*. Of course, *util* has no physical substance, but they serve as units of measurement of utility. The assignment of a quantity to the utility that a person derives is the function of the *individual's utility function*. According to Game theory, even when they are not consciously aware of it, people strive to maximize the utility function associated with themselves [3, 5]. This, in many cases, coincides with other objectives that are readily apparent [6].

Game theory is the ultimate modeling system for security systems and, more recently, cybersecurity. It allows the creation of tangible solutions that will enable the evaluation of existing strategies and their optimization to create a robust and long-term security environment at the organization level [7]. Using the principles of game theory [8], it is possible to develop cyber-threat scenarios where cyber security professionals can apply the strategies that govern the organization and control or quantify the risk to their valuable assets [1]. They can also use areas with a low level of risk to maximize the return on their investments. As a result, using specialized scenarios based on game theory, it is possible to predict the attackers' strategy at each stage of the attack cycle, assisting in developing intelligent models to improve cybersecurity and creating new intelligent systems to defraud the attackers [9].

Nevertheless, the inherent difficulty of implementing the proposed solutions that may arise from performing tests—simulations based on game theory, lies in the combined explosion of all possible combinations that make up the solution space, the entire examination of which requires a considerable computer time and corresponding computing resources, to the point that their use becomes unprofitable [5]. This weakness is because, even for minor problems, the possible strategies available to the defender are finite but at the same time numerous. For this reason, the work proposes a hybrid system [10, 11], which aims to identify the best-possible solutions in the area of theoretically optimal solutions in a short time and with minimal computing resources. Specifically, a heuristic optimization [12, 13] methodology is used with overlapping solutions between two contiguous neighborhoods based on the Bloom Filters structure that supports fast listings and searches [14, 15]. This methodology, which has been tested in optimizing safety strategies in the sports industry, achieves up to 40% optimization compared to other methods.

## 2. Relevant Publications

Artificial intelligence in cybersecurity is a concept that is continuously evolving. The literature on decision-making, deep learning, and game theory focuses on utilizing different concepts to efficiently solve complex real-world cybersecurity problems [3, 16, 17].

Das and Sandhane [18] offered a concise summary of AI applications of major cybersecurity solutions and assessed the potential for boosting cybersecurity capabilities via defensive mechanism enhancements. To begin, neural networks are utilized for safeguarding the periphery and a variety of other protection domains. On the other hand, it was evident that some cybersecurity issues could be resolved effectively only via artificial intelligence technologies. For example, thorough intelligence is critical for strategic decision-making, and logical judgment support is one of the unresolved protection concerns. While neural networks were not the best technology for many applications, advanced cybersecurity measures remained necessary. These domains included decision support, situational awareness, and data access.

Johnson [19] examined the influence of AI on strategic stability, focusing on the dangers and trade-offs associated with predelegating military power (or automating aggression) to robots. He contended that AI-enabled decision-support tools—supplanting human analytical reasoning, compassion, inventiveness, and imagination in the strategic judgment method—would be profoundly disastrous if defense planners came to view AI support' function as a magic bullet human assessment and decision-cognitive making's inadequacies. Additionally, the article discussed the malicious use of artificial intelligence-enhanced fake reports, deepfakes, bots, and other forms of social media by nonstate actors and state proxy actors, which may produce states to overestimate a threat by unclear or exploited information, thereby growing destabilization.

Alpcan and Basar [20] systematically sought to provide a conceptual framework for making resource allotment choices that balance existing skills and perceived security issues in their literature about network security and game-theoretic techniques. They concentrated on applying game, data, interaction, efficiency, selection, and control theories to various security difficulties. Simultaneously, links between conceptual models and real-world security issues are emphasized to generate a key review loop between principles and application.

Nguyen and Reddi [21] investigated the application of Deep Reinforcement Learning (DRL) approaches in cyber warfare. They explored a variety of vital subjects, including DRL-based defense techniques for cyber-physical assets, autonomous intrusion discovery, and multiagent DRL-based game theory simulations for cyberattack defense measures. Furthermore, comprehensive arguments and possible research directions on Internet security emphasize DRLs are offered. They hoped that this extensive review would provide a framework for and motivate further research into the capability of developing DRL to deal with increasingly advanced digital confidentiality complications.

Schlenker et al. [8] investigated the fundamental inherent problem of assigning cyber warnings to a small number of security experts. They investigated this issue using the Cyber-alert Allocation Game and demonstrated how to compute the defender's best options. They proposed a novel method for dealing with concerns about implement ability when determining the defender's most acceptable marginal technique to overcome this game. Finally, they provided heuristics for resolving large games similar to those depicted, and an objective assessment of the methodology and treatment approaches proposed.

## 3. Definition of the Problem

Even for a system under study related to the sports industry, the security of which requires the design of a system of strategies, specific decisions need to be identified [7, 19]. Let (*a*_1_, *a*_2_,…, *a*_*n*_) be the vector of the above decisions, which are also called design variables, and can be any system design security decisions. The security engineer is asked to decide the best possible system design [22], given the constraints imposed by the nature of the system, security policies applied, system functionality, users, etc. It should be noted that the variables can differ depending on their nature, the point of danger presented by change, and the problem that the security engineer is called to solve; for example, the problem can be size optimization, topology optimization, system optimization, financial optimization, and so on [23]. With the proposed method, it is possible to use game theory to solve these problems and, at the same time, identify combinations thereof [9].

**Figure 1:**
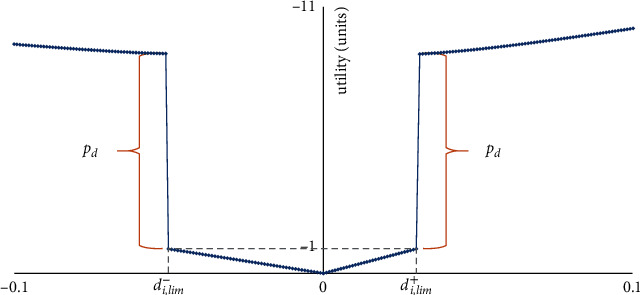
Travel scenario utility function.

The problem can be analyzed in two substages: the construction of the player's earnings registers and the analysis of the game to select the best strategy [24] for the defending player (i.e., the security engineer). Specifically, the first step, in particular, forecasts what will occur in each case of travel planning (as travel is considered as the operating cost for each action, with a positive direction as the tolerable cost while a negative approach is the opposite) [1, 25, 26].  Figure 1 shows the travel benefit function.

The mathematical expression of the scenario examined, as shown in Figure 1 is [2, 4, 26]:

(1) $$\begin{array}{c} u_{d,i}\left(d_{i}\right)=\left\{\begin{array}{c} a_{d,i}^{+}\cdot d_{i},\;0\leq d_{i}\leq d_{i,lim}^{+},\\ a_{d,i}^{-}\cdot \left|d_{i}\right|,\;d_{i,lim}\leq d_{i}<0,\\ a_{d,i}^{+}\cdot d_{i,lim}^{+}+p_{d}+a_{d,i}^{+}\cdot {\left(d_{i}-d_{i,lim}^{+}\right)}^{k},\;d_{i}>d_{i,lim}^{+},\\ a_{d,i}^{-}\cdot \left|d_{i,lim}^{-}\right|+p_{d}+a_{d,i}^{-}\cdot {\left(d_{i,lim}^{-}-d_{i}\right)}^{k},\;d_{i}<d_{i,lim}^{-}, \end{array}\right) \end{array}$$

*u*_*d*,*i*_(*d*_*i*_): is the utility function for a particular travel *d*_*i*_, *d*_*i*,*lim*_^+^: is the limit of travel of a degree of freedom in a positive travel, which is desirable not to be exceeded; *d*_*i*,*lim*_^−^: is the limit of travel of any degree of freedom in the negative direction, which is desirable not to be exceeded; *p*_*d*_: is the penalty imposed if one of the travel limits for this degree of freedom is surpassed. Its price is negative, *a*_*d*,*i*_^+^: a coefficient showing the linear change of the benefit for small values of the *d*_*i*_ travel when it has a positive direction. It is strictly negative and indicates the preference of player *A* for more minor travels than larger ones, *a*_*d*,*i*_^+^: coefficient showing the linear change of the benefit for small values of the *d*_*i*_ movement when it has a negative direction. It is strictly negative and indicates the preference of player *A* for smaller movements over larger ones, *k*: indicator showing how the utility changes for *d*_*i*_ movement values greater than the corresponding desired limit.

Respectively, the following function determines the benefit due to trends (as trends are considered the operating cost for each possible action, with a positive direction indicating the modest cost and a negative direction meaning the opposite) [27–29]:

(2) $$\begin{array}{c} u_{\sigma ,i}\left(\sigma_{i}\right)=\left\{\begin{array}{c} a_{\sigma ,i}^{+}\cdot \sigma_{i},\;0\leq \sigma_{i}\leq \sigma_{i,lim}^{+},\\ a_{\sigma ,i}^{-}\cdot \left|\sigma_{i}\right|,\;\sigma_{i,lim}^{-}\leq \sigma_{i}<0,\\ a_{\sigma ,i}^{+}\cdot \sigma_{i,lim}^{+}+p_{\sigma }+a_{\sigma ,i}^{+}\cdot {\left(\sigma_{i}-\sigma_{i,lim}^{+}\right)}^{k},\;\sigma_{i}>\sigma_{i,lim}^{+},\\ a_{\sigma ,i}^{-}\cdot \left|\sigma_{i,lim}^{-}\right|+p_{\sigma }+a_{\sigma ,i}^{-}\cdot {\left(\sigma_{i,lim}^{-}-\sigma_{i}\right)}^{k},\;\sigma_{i}<\sigma_{i,lim}^{-}. \end{array}\right) \end{array}$$

The overall benefit of the results is summarized in the equation:

(3) $$\begin{array}{c} u_{res}=w_{d}\cdot u_{d}+w_{\sigma }\cdot u_{\sigma }, \end{array}$$

where

(4) $$\begin{array}{c} u_{d}=\sum w_{d,i}\cdot u_{d,i},\\ u_{\sigma }=\sum w_{\sigma ,i}\cdot u_{\sigma ,i}. \end{array}$$

The sizes *w*_*d*_ and *w*_*σ*_ express the importance of movements and trends, respectively, in shaping the usefulness of the results. If restrictions are placed on only one of the two types of outcomes of interest, then this takes on total weight of 1 while the other is 0.

Respectively, the quantities *u*_*d*_ and *u*_*σ*_ express the gravity coefficients of the individual movements and stresses, i.e., the importance of any node movement or trend that develops in a member in the final utility configuration [23, 30]. For these rates, we have

(5) $$\begin{array}{c} \sum w_{d,i}=1,\\ \sum w_{\sigma ,i}=1. \end{array}$$

The value of the travel or trend factor is determined by whether the node is committed to the specific movement or whether the particular member has a limit on the trend that develops. Thus, if the total number of travel constraints is denoted by *n*_*c*,*d*_ and the number of constraints on trends by *n*_*c*,*σ*_, then the individual importance factors will take values:

(6) $$\begin{array}{c} \begin{array}{c} w_{d,i}=\left\{\begin{array}{c} \frac{1}{n_{c,d}},\\ 0, \end{array}\right)\;\\ w_{\sigma ,i}=\left\{\begin{array}{c} \frac{1}{n_{c,\sigma }},\\ 0. \end{array}\right) \end{array} \end{array}$$

The preferences of player *A* must also consider the fact that in addition to smaller transfers that are equivalent to the minimum operating cost, it must also seek the lowest possible financial cost. Therefore, it is necessary to determine the utility that it derives due to the financial cost. This is defined as [9, 17, 31, 32]:

(7) $$\begin{array}{c} u_{de\;s}=\left\{\begin{array}{c} a_{\text{cost}}\cdot \text{cost},\text{ cost}\leq \text{budget,}\\ a_{\text{cost}}\cdot \text{cost}+p_{\text{budget}},\text{ cost}>\text{budget,} \end{array}\right) \end{array}$$

where cost is the total cost of the security plan, *a*_cost_ is a negative factor that indicates the preference of player *A* for cheaper modes of action than more expensive, and *p*_budget_ is a penalty imposed if a permissible cost limit is exceeded.

The overall benefit enjoyed by player *A* will result from the simultaneous action of the benefits due to design and results. The degree to which each affects the result depends on the user and is what will largely determine the result of the optimal strategy:

(8) $$\begin{array}{c} u_{A}=w_{res}\cdot u_{res}+w_{de\;s}\cdot u_{de\;s}, \end{array}$$

where *w*_*res*,_*w*_*de*  *s*_ are the weights that the user assigns to the analysis and design results, respectively. Of course, they must satisfy the property:

(9) $$\begin{array}{c} w_{res}+w_{de\;s}=1. \end{array}$$

In the usual case, the importance and the results of the analysis are given equal importance, so the rule is

(10) $$\begin{array}{c} w_{res}=w_{de\;s}\\ =\frac{1}{2}. \end{array}$$

The utility function of player B, which requires action as it creates security incidents, is taken equal to and opposite to the utility function of player *A* so that the benefit of one player is to the detriment of the opponent:

(11) $$\begin{array}{c} u_{B}=-u_{A}. \end{array}$$

The earnings of player *A* for each mitigation system design and each decision form the register of the game's profits, from the solution of which will emerge the best-defense strategy of the system in each phase of the attack [9, 20, 33]. We assume that the players' utility functions are equal and opposite for this modeling, which places the game in the category of zero-sum games. In this type of game, each outcome is antagonistic for both players, in the sense that what one player “wins,” the other “loses.”

## 4. Optimization

In the previous section, we developed a methodology for solving security solution design problems. Mathematical optimization involves selecting the optimal component concerning a given criterion from a given group of potential solutions. Problems relating to optimization appear in each of the quantitative subfields, including computer science and engineering, as well as operations research and economics. An optimization problem can be simplified to its most basic form by stating that its solution is to maximize or minimize an objective function. This can be accomplished by methodically selecting input values from within an allowable set and computing the value of the process. A significant portion of applied mathematics uses optimization theory and methods for different formulations. In a broader sense, optimization refers to determining the “best available” values of a particular objective function given a specific domain (or input). This process might involve various objective functions and several different disciplines.

Solution extraction consists of finding the line of the usual form table of the corresponding game with the largest column minimum [7, 34]. To make this possible, the standard game board must be constructed. All possible design options the defender can choose (strategies) must be listed and analyzed for each possible decision case, and then the utility table and the game in its standard form must be developed to follow the resolution process [35, 36].

The above process presents significant computational issues [37–39]. When the construction has *m* groups of members, *N*^*m*^ possible choices for each member and *L* possible decision-making cases, the possible designs are *N*^*m*^, and the static analyzes to be performed are *N*^*m*^ × L. This size is too large even for medium-sized problems. For example, if *m* = 8, *N* = 20, *L* = 5, then a total of 20^8^ × 5 = 1.28 × 10^11^ static analyzes must be performed. When one static solution takes around 0.015 seconds to execute, the total analysis time is 1.28 × 10^11^ × 0.015 = 1.92 · 10^9^ seconds, i.e., about 61 years.

On the other hand, even if the analysis time is reduced, the algorithm's complexity remains enormous. The number of member groups is the most decisive factor, as it exponentially increases the size of the problem. For example, if the member groups in the above scenario increase to 9 instead of 8, the estimated solution time will exceed 1200 years. However, a mediocre computer will face memory problems in addition to the problem of resolution time, as it is required to store logs with a large number of data, making it impossible to solve the problem in this manner. So, as it is understood, the need for a faster solution is imperative, so there is a shift to heuristic algorithms [7, 9, 40].

**Figure 2:**
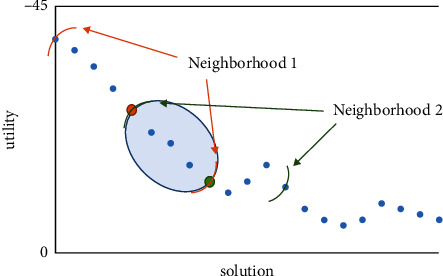
Overlap of solutions between two contiguous neighborhoods.

The heuristic algorithm presented in this paper is based on the observation that at each step, most of the bioinspired heuristic algorithms define an area in which, after their analysis, they find the best solution, with the center forming a new neighborhood. However, when examining the new neighborhood, previously rejected solutions are re-examined. At best, half of the solutions of the previous neighborhood are re-examined [41–44]. This overlap of solutions is typically illustrated in Figure 2.

The above shows that the algorithm is called upon to repeat calculations for cases it has already considered in the previous steps. In many cases, this extra work is less laborious, as it is enough to simply calculate the candidate solution's cost. In other cases, however, the cost passes the first checkpoint of the algorithm. It is followed by static resolution and determination of the benefit, a more complicated process. In any case, the algorithm is procrastinating for no reason.

To address this flaw, it is reasonable to keep a record of the solutions that have been tested so that their analysis is not repeated in the subsequent steps. This record has three crucial features [7, 13, 37]:

1. The number of solutions that will be recorded will be pretty large and therefore using the typical list will consume a significant part of the computer memory.
2. The number of recordings cannot be determined in advance. This number can be estimated, but not accurately. This means that the number of positions in a list that includes them cannot be determined.
3. The registration and search process for verification may not be fast enough.

This paper uses bloom filters to solve all of these problems.

## 5. Bloom Filters

Bloom Filters [45] are data structures that have an advantage over other data lists. They are more efficient in memory space, allowing for a chance of error when searching within logs, offering significant gains in complex applications [34, 46]. The structure of a Bloom Filter supports very fast listings and searches. Their most important advantage lies in their much better performance in a large volume of data and the minimal memory space used per registered object. Their use is ideal for applications in which one wants to check if a value is included in a list, but for bloom filters to make sense, it must first be demonstrated that there is a possibility of limiting the likelihood of error [12, 47, 48]. The more memory space available, the more this possibility will tend to be eliminated. Also, a key issue is the determination of the appropriate parameters to optimize the efficiency of the bloom filter, i.e., to achieve low-memory usage with an acceptable error rate at the same time [45, 49].

The following assumption is made for the proposed implementation: all matching mechanisms operate in a completely random manner. This means that the positions (in series of bits) identified by matching mechanisms acting on a specific object follow a uniform distribution and are entirely independent of the other entries. Given the use of *n* bits and the input of a set of data *S*, set *s*, it is desirable to determine the error probability. A question that arises is, what is the expected number of values of “1” after introducing all objects. To resolve this, a specific position in the string of bits will be examined. The probability that the value 1 will exist after the entries are in that position will be calculated [37, 50].

Initially, the probability that this bit will have the value 0 after the entries will be determined. The probability of getting the value 1 by the action of a matching mechanism is only 1/*n*, so the probability of staying 0 is (1 − 1/*n*). Therefore, for *k* matching mechanisms and *s* number of objects, the chance becomes (1 − 1/*n*)^*k*·*s*^ and finally, the probability for each bit to be set “1” after all entries will be [36, 44, 51, 52]

(12) $$\begin{array}{c} 1-{\left(1-\frac{1}{n}\right)}^{k\cdot s}. \end{array}$$

**Figure 3:**
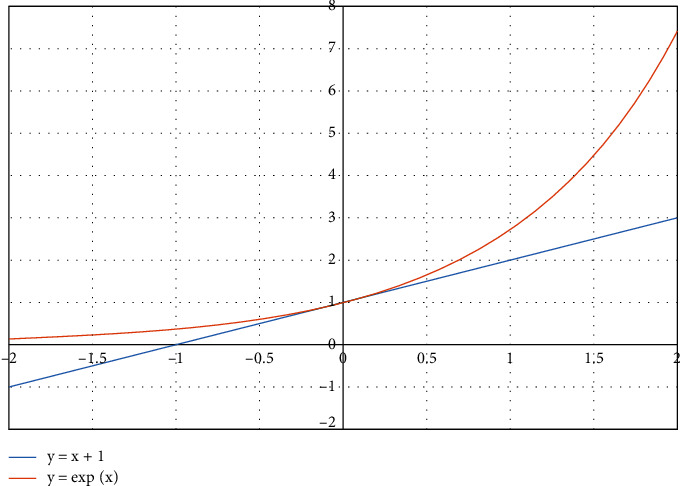
The functions *y*_1_=1+*x*, *y*_2_=*e*^*x*^.

Observing Figure 3, it is evident that the function *y*_1_=1+*x* is upper blocked by *y*_2_=*e*^*x*^ since *e*^*x*^ ≥ 1+*x*,  ∀ *x* ∈ *R*, while for *x*⟶0⇒*e*^*x*^≃1+*x*.

Therefore, the above probability, respectively, will apply:

(13) $$\begin{array}{c} 1-{\left(1-\frac{1}{n}\right)}^{k\cdot s}≃1-e^{-k\cdot s/n}, \end{array}$$

and because *b*=*n*/*s*, where *b* are the bits per object, eventually the probability is configured as follows:

(14) $$\begin{array}{c} 1-{\left(1-\frac{1}{n}\right)}^{k\cdot s}≃1-e^{-\left(k/b\right)}. \end{array}$$

From this relationship, it is now clear that as the number of bits per object increases, the probability of a particular bit becoming “1” tends to zero.

Now, for example, an object *ε*, *x* ∉ *S* is examined. To make a mistake, all *k* bits in the corresponding positions must have the value 1, so this probability is [9, 47, 53, 54]:

(15) $$\begin{array}{c} \varepsilon ≃{\left[1-e^{-\left(k/b\right)}\right]}^{k}. \end{array}$$

So in order to determine parameters that result in a small tolerable error while using a small memory space, the number of bits per object must be determined, i.e., *b*. For given *b*, the error *ε* turns to be minimized for a value of *k*≃ln2 · *b*. *k* must be an integer, extracted after rounding the above number. This is how it turns out [47, 55, 56]:

(16) $$\begin{array}{c} \varepsilon ≃{\left(\frac{1}{2}\right)}^{\text{ln}2\cdot b}. \end{array}$$

This equation can be expressed in terms of *b*, so for a given error, the bits per entry are calculated:

(17) $$\begin{array}{c} b≃1.44\cdot {\text{log}}_{2}\left(\frac{1}{\varepsilon }\right). \end{array}$$

For example, for 8 bits per entry is calculated:

(18) $$\begin{array}{c} k≃\text{ln}2\cdot 8≃5.54\Rightarrow k=5. \end{array}$$

And it turns out:

(19) $$\begin{array}{c} \varepsilon ≃2\text{\%}. \end{array}$$

Indicatively, it is stated that if the bits per entry are doubled to 16, then this probability becomes approximately:

(20) $$\begin{array}{c} \varepsilon ≃0.4‰. \end{array}$$

## 6. Conclusions

The inherent difficulty of applying the proposed security solutions to cybersecurity problems lies in the situations characterized by the combined explosion of all possible combinations that make up the solution space. Developing and finding the optimal solution takes a significant amount of computational time and computing resources to the point where their use is frequently unprofitable, especially in high-risk rearranged environments. This weakness exists because, even for minor issues, the defender's available strategies are limited but numerous. For this reason, the paper proposed a hybrid system of heuristic intelligent algorithms, which aims to identify, in a short time and with minimal computing resources, the best-possible solutions in the area of the theoretically optimal solution. As it turned out experimentally, by utilizing bloom filters, the system significantly reduced computing time and the corresponding required resources.

The bloom filters are both in complex applications and in a simple use such as the one that the improved gradual impairment algorithm is called to do. For this problem, some remarks are made [34, 39, 57, 58]:

1. It has already been stated that we insert values into a bloom filter rather than items. On the other hand, each potential answer is fundamentally a list, or more specifically, an object that includes the serial numbers of the individual member groups. The utilization of this solution's serial number enables the execution of the procedures mentioned earlier. Because of this, the bloom filter will enter the serial number of the resolution, which is going to be different for every key, and then based on this. The entries are going to be searched again.
2. The little chance of making a mistake, which is already very low, is not a cause for concern because it is highly improbable that this will result in a significant error. In particular, when the bloom filter is looking for a solution, it may believe that it already exists in its list. As a result, the answer will not be subjected to further analysis. It would be a terrible turn of events if this (very uncommon) instance of wrongfully rejected evidence turned out to be the best.
3. The bloom filter was applied to the issues that arose and provided conclusive evidence of its speed and general utility. To be more specific, its use prevents the study of 20–40% of the total solutions that were initially investigated, which results in a proportional reduction in the amount of time needed for computing.

There are some disadvantages to using bloom filters in specializing and evaluating the experimental process. Specifically, we cannot register objects, nor pointers to objects, only values. So, during the search, a check is made whether a value has been met or not in the registration phase. A second disadvantage is that deletions from the entries are not allowed. Finally, the most critical weakness is the possibility of error. If a value has been entered, there is no way it can be mistakenly considered that it is not included in the entries. On the contrary, there is a particular possibility that a value has not been recorded, and the bloom filters during the search process claim that it has been found in the list. These drawbacks are also research questions that will be addressed in future extensions of this work.

## Data Availability

The data used in this study are available from the author upon request.
